# RE: learning medical professionalism – the application of appreciative inquiry and social media

**DOI:** 10.1080/10872981.2020.1780723

**Published:** 2020-06-19

**Authors:** Mohsin Abedi, Aqsa Khurram, Dina Abedi

**Affiliations:** a Faculty of Medical Sciences, Newcastle University, Newcastle-Upon-Tyne, UK; b Barts and the London School of Medicine and Dentistry, London, UK

**Keywords:** Medical education, medical professionalism, social media, SNS’s, appreciative inquiry

## Abstract

A letter to the editor in response to the article by Hsieh et al. published in this journal, discussing the application of appreciative inquiry and social media on medical professionalism education.

Dear Editor,

Firstly, we would like to thank Hsieh et al. [1] for their insightful and necessary exploration into the application of appreciative inquiry models and social media, and its impact on medical professionalism teaching. As senior medical students interested in education, the article certainly piqued our curiosity.

Indeed, the authors state that qualities encompassed by the notion of medical professionalism are often intangible and, therefore, difficult to measure. This is an excellent point- medical education is becoming profoundly centred on principles of clinical competence and rigour [2]. Medical professionalism is an anomaly in that it does not yet have a global, objective and transferrable code which educators and observers may use to evaluate learners. As students, we can testify to this; we seldom receive feedback on our professionalism, with checklists relating to clinical procedures often favoured in our curriculums.

We wish to praise the commitment of the authors to digitisation in the context of medical education, as well as the identification of social media as a potential platform for it. This innovative implementation of social media platforms may revolutionise medical education moving forward; particularly given the repercussions of Covid-19, and the newfound emphasis on remote service delivery.

We are intrigued by the interesting and nuanced comparison by Hsieh et al. of far-Eastern and traditional Western medicine approaches to medical professionalism, especially the differences in rootedness of the importance given to role-models. This discussion culminates in the question posed by the authors: ‘can Western medical professionalism education be directly transferred to other cultural areas without causing conflict or being questioned?’. We argue that ‘culture-clashes’ cannot be assumed to be solely dependent on differences in medical professionalism ideology, with other factors at play.

The authors go on to suggest that education content and scope should be adjusted according to culture. We argue, however, that in order to achieve uniformity, we must come up with an international standard. Cross-cultural differences do exist, and result in various discrepancies in the interpretation of medical professionalism [3]. In this globalised and digital world, uniformity may enable seamless transitions for medical professionals working cross culturally, and between care systems.

The importance given by the authors to teachers and role models in the process of medical professionalism education provision is interesting. Behavioural studies have demonstrated the significance of observational learning, highlighting the apparent innateness of humankind’s tendencies to ‘do as we see’ [4], which further strengthens the authors’ point that this can be used as a tool for teaching medical professionalism. We also feel the model proposed by the authors allows educators to be made aware of their shortcomings and successes as pedagogues, which is a vital characteristic for not only educators, but all medical professionals to have. Reflective practice ‘is a paper requirement’ for one’s progression through healthcare [5]. However, the reliance placed on role models increases the chances of the propagation of poor practice habits if misunderstood by the student, as well as the potential pressure teachers may place on students to skew study results to depict them more favourably.

This research is limited by the fact that all students are first year clinical students. The implication of this is that, due to a limited exposure to clinical settings and practices, the students involved may not have had the required experience to accurately and reliably appraise educators. As medical students, we know it is difficult without adequate experience to have a strong grasp of professionalism and good practice- so it would be both unfair and dangerous to rely on such students to draw such conclusions, or report, on these things. Further, we feel a high degree of subjectivity and, at the same time, a lack of scrutiny of the actual application of mandated professionalism principles, means there could be both misplaced positive reinforcement of bad practice, and inappropriate adjustment of self (by both teachers and students)- despite the efforts of the authors to mitigate the risks of these occurring.

The methodology itself leans a great deal on social media. While we praised social media as a potential platform for education, it must be stated that social media has a great capacity to influence others. The visibility of all responses to all participating students increases the likelihood of students being encouraged or discouraged to post certain evaluative comments.

Moving forward, the models used by the authors can certainly be used, with appropriate revisions, to provide not only medical professionalism teaching to students, but teaching which covers a range of subspecialties and modules within clinical medicine. The authors’ model, in conjunction with an international standard, can ensure there is no more ambiguity regarding what it means to be ‘professional’, and that medical professionalism can be better observed, evaluated and taught. We must do all that we can to ensure medical professionals are on the same page regarding medical professionalism, regardless of location.
